# Exploring the impacts of risk factors on mortality patterns of global Alzheimer’s disease and related dementias from 1990 to 2021

**DOI:** 10.1038/s41598-024-65887-4

**Published:** 2024-07-06

**Authors:** Tofigh Mobaderi, Anoshirvan Kazemnejad, Masoud Salehi

**Affiliations:** 1 Department of Biostatistics, Faculty of Medical Sciences, Tarbiat Modares University, Tehran, Iran; 2 Nutritional Sciences Research Center, Iran University of Medical Sciences, Tehran, Iran; 3 Department of Biostatistics, School of Public Health, Iran University of Medical Sciences, Tehran, Iran

**Keywords:** Alzheimer’s disease, Dementia, Clustering, Longitudinal k-means clustering, Generalized estimating equations (GEE), Neurological disorders, Risk factors

## Abstract

Alzheimer’s Disease and Related Dementias (ADRD) affect millions of people worldwide, with mortality rates influenced by several risk factors and exhibiting significant heterogeneity across geographical regions. This study aimed to investigate the impact of risk factors on global ADRD mortality patterns from 1990 to 2021, utilizing clustering and modeling techniques. Data on ADRD mortality rates, cardiovascular disease, and diabetes prevalence were obtained for 204 countries from the GBD platform. Additional variables such as HDI, life expectancy, alcohol consumption, and tobacco use prevalence were sourced from the UNDP and WHO. All the data were extracted for men, women, and the overall population. Longitudinal k-means clustering and generalized estimating equations were applied for data analysis. The findings revealed that cardiovascular disease had significant positive effects of 1.84, 3.94, and 4.70 on men, women, and the overall ADRD mortality rates, respectively. Tobacco showed positive effects of 0.92, 0.13, and 0.39, while alcohol consumption had negative effects of − 0.59, − 9.92, and − 2.32, on men, women, and the overall ADRD mortality rates, respectively. The countries were classified into five distinct subgroups. Overall, cardiovascular disease and tobacco use were associated with increased ADRD mortality rates, while moderate alcohol consumption exhibited a protective effect. Notably, tobacco use showed a protective effect in cluster A, as did alcohol consumption in cluster B. The effects of risk factors on ADRD mortality rates varied among the clusters, highlighting the need for further investigation into the underlying causal factors.

## Introduction

Alzheimer’s Disease and Related Dementias (ADRD) are progressive neurodegenerative disorders that lead to memory loss, cognitive decline, and behavioral changes^1,2^. ADRDs are a group of complex brain illnesses that begin with mild memory loss symptoms and eventually become severe enough to interfere with daily tasks^2^. The rising incidence rates of Alzheimer’s disease and other types of dementia have led to increasing public health problems due to the rising global population ages and life expectancy, resulting in different consequences for individuals, families, healthcare systems, and society^3^.

According to reports, the global number of people with Alzheimer's disease and related dementias increased to 51.62 million in 2019, indicating a significant increase of 161% compared to 19.79 million in 1990^4^. It is estimated that by 2050, the number of affected individuals will reach 152.8 million^5^. It is well documented that the mortality rates of ADRD are heterogeneous across geographies. In Europe, approximately 9.1 million individuals were identified with dementia in 2018^6^. This number is estimated to increase to 16.8 million people by 2050^6^. Dementia presents a significant public health issue in countries with low and moderate-income levels^7^. In 2010, 58% of people with dementia lived in these countries, and it is projected that this proportion will rise to 63% by 2030 and further increase to 71% by 2050^7^. Dementia cases are expected to increase in every country, with the smallest estimated increases in high-income Asia Pacific (53%) and Western Europe (74%). On the other hand, the largest growth is anticipated in North Africa and the Middle East (367%) and eastern sub-Saharan Africa (357%)^8^. In 2019, dementia was the seventh leading cause of death globally, responsible for 17.3% (1.6 million) of neurological deaths^9,10^. Among individuals aged over 70 years, it ranked as the fourth leading cause of death^10^.

Since there are currently no available treatments to cure or change the progressive nature of ADRD, it is crucial to identify risk factors that can be modified in order to decrease the occurrence of the disease, delay its onset, or minimize its impact^1^. Previous studies have shown that certain risk factors, such as age, gender, family history, smoking, alcohol consumption, cardiovascular disease, diabetes, less education, obesity, and physical inactivity, are associated with the incidence of ADRD^11,12^. Although some risk factors, such as age and family history, cannot be changed, there are other risk factors that can be modified to reduce the risk of cognitive decline and dementia. By modifying these risk factors, it is possible to prevent or delay up to 40% of dementias, which presents important opportunities for prevention and intervention^11^.

Analyzing the growth trajectory of ADRD death rates over time can provide valuable insights for policymakers, enabling them to identify and investigate the risk factors associated with the disease. There are several statistical methods that can be used to analyze the longitudinal pattern of the ADRD mortality rate over a specific time period. Generalized Estimating Equations (GEE) is a robust statistical approach for modeling longitudinal data that takes into account the correlation between data points^13^. By considering the correlation structure, it allows for a comprehensive analysis that captures the relationships and dependencies within the dataset, enhancing the accuracy and reliability of the results^14^. However, the GEE model estimates the overall mean and does not take into account the heterogeneity among the longitudinal patterns of the data. Neglecting the possible variations in the patterns and focusing on the overall trend can result in misunderstandings and incorrect conclusions regarding the effects of covariates and the underlying growth process of the outcome variable^15^. Hence, it is important to categorize the data into homogenous groups to gain a more accurate understanding of the individual trajectories and the factors influencing them. Longitudinal k-means clustering is a methodology used in data analysis and pattern recognition to group similar trajectories over time^16^. It is an extension of the traditional k-means clustering algorithm that is used for longitudinal or time-series data. In longitudinal k-means clustering, the sequential similarity nature of data points that were collected over multiple time points would be considered^16^. It aims to group similar trajectories or patterns based on their temporal evolution rather than just their static characteristics.

In our literature review, we were unable to find any published research that specifically examines the patterns of ADRD mortality rates while considering the heterogeneity among countries across the globe. Therefore, the objective of this study was to cluster the overall mortality rate patterns of Alzheimer's and other dementias worldwide from 1990 to 2021. Subsequently, we used a GEE model to estimate the effects of risk factors in each cluster on men, women, and the overall ADRD mortality rates. The results of the study may help to improve our understanding of the patterns in ADRD mortality rates. Additionally, the classification of countries based on their growth trajectories can provide valuable insights into identifying potential risk factors and serve as a roadmap for guiding public health interventions.

## Methods

In this longitudinal study, mortality rate data for Alzheimer’s Disease and Related Dementias (ADRD) per 100,000 population were obtained for 204 countries and territories in 1990, 1995, 2000, 2005, 2010, 2015, and 2021 from the Global Burden of Disease (GBD) platform^17^. These data were extracted for men, women, and the overall population, and they were considered the main outcome of the study. Along with these data, information on various other risk factors was extracted from different sources for the same time points (from 1990 to 2021). These risk factors were considered as covariates and will be described as follows: The time variable was considered as a covariate and recoded as 1 to 7 for the years 1990 to 2021, respectively. Cardiovascular disease prevalence and diabetes prevalence were extracted from GBD, and the Human Development Index (HDI) and Life Expectancy at Birth (LEB) were obtained from the United Nations Development Programme (UNDP) platform^17,18^. The LEB is a standard metric that estimates the average lifespan of a newborn, assuming that current age-specific mortality rates remain constant throughout their life^19^. The HDI is a composite index that includes LEB, expected years of schooling, and Gross National Income (GNI) per capita. This index ranges from 0 to 1 and provides a measure of a country's overall development level^20^. In addition, the consumption of alcohol and the percent of estimated tobacco use prevalence were obtained from the World Health Organization (WHO) platform^21,22^. Total alcohol consumption per capita is defined as the total volume of alcohol consumed per adult (15 years and older) in a calendar year, expressed in liters of pure alcohol and adjusted for tourist consumption^21^. The tobacco use prevalence and alcohol consumption data were unavailable for 1990 and 1995. As a solution, we substituted the data from 2000 for these two years. Additionally, the alcohol consumption data for 2021 was substituted with the data from 2019, as it was not available specifically for 2021. All the mentioned data was extracted for men, women, and the overall population. It is important to note that the HDI, cardiovascular disease prevalence, tobacco use, and diabetes prevalence, which range from 0 to 1, are multiplied by 100 to change the scale to 0 to 100 for easier interpretation in statistical analysis.

Descriptive statistics, including mean and standard deviation, were calculated for all variables at each time point to provide a comprehensive overview of the data. Additionally, Spearman correlation coefficients were computed to examine the relationships between all variables. Subsequently, the statistical analysis started with five different Generalized Estimating Equations (GEE) models conducted to estimate the overall effect of time and other covariates on the ADRD mortality rate of men, women, and the overall population across 204 countries and territories from 1990 to 2021. In the first Model, the time variable was included as a covariate to estimate the pattern of ADRD mortality rate during the study period. Subsequently, cardiovascular disease and diabetes were added to Model 2. For Model 3, the HDI and LEB were included, and then the alcohol and tobacco covariates were added to Model 4. In Model 5, due to the unexpected and suspicious effects of alcohol and tobacco on the ADRD mortality rate, we decided to include interaction terms for alcohol and tobacco with HDI to investigate potential interaction effects.

In the second step, the longitudinal k-means clustering approach was employed to explore the heterogeneity among countries based on their ADRD mortality rate patterns from 1990 to 2021. The longitudinal k-means clustering approach is a non-model-based and non-parametric algorithm that does not require any prior assumptions^16^. The appropriate number of clusters was determined using the Calinski–Harabasz index, with the highest value of the index indicating the suitable number of clusters^16^. The longitudinal k-means clustering approach was utilized on the overall data to classify countries according to their patterns of ADRD mortality rates. The same clusters derived from the analysis of the overall data were applied to both men and women, enabling a comparison of risk factor effects between the two genders. The Calinski–Harabasz criterion was calculated for models with 2 to 5 clusters, and the model with the highest criterion was selected. The decision to limit the clusters to a maximum of 5 was made to avoid decreasing sample sizes in subgroups, which would hinder the estimation of risk factor effects on subgroup mortality rates using GEE models.

Finally, the GEE model was used to estimate the effects of risk factors on the trend of the ADRD mortality rates in each cluster for men, women, and the overall population. The GEE model selection for the clusters was the same as the GEE model used for the total data, except for model 5, which was excluded due to interaction terms. We chose not to include interaction terms in modeling the clustered data because clustering categorizes the data into homogeneous samples with similar patterns. Therefore, we expected that clustering would naturally reduce the potential for interactions. The longitudinal k-means clustering was performed using the kml package in R software, and the GEE model was conducted using SPSS 26 software. For this study, a p-value of 0.05 or lower was deemed statistically significant. The geographic distribution map of countries and territories worldwide was generated using ArcGIS 10.8 software. Furthermore, AI assistance tools like OpenAI ChatGPT and Grammarly were utilized in this study to perform tasks such as text translation, clarity correction, summarization, and grammar correction^23,24^. It is important to note that these tools were employed to enhance the precision and grammatical accuracy of the text, rather than to generate content.

## Results

The descriptive statistics, including the mean and standard deviation (SD), of all the study variables for men, women, and the overall population from 1990 to 2021, are reported in Table 1. The mortality rates of Alzheimer’s Disease and Related Dementias (ADRD) across 204 countries and territories for men, women, and overall data were 7.78, 15.12, and 12.21 persons per 100,000 population in 1990, respectively. These rates steadily increased to 15.26, 31.56, and 23.54 persons per 100,000 in 2021, respectively. Additionally, the percentage of cardiovascular disease prevalence, diabetes prevalence, HDI, and LEB increased during the study period. However, alcohol consumption remained relatively constant, while tobacco use decreased from 1990 to 2021. The mortality rates of ADRD and LEB were higher in women compared to men. However, in contrast to women, men had higher HDI, alcohol consumption, and prevalence of tobacco use.

**Table 1:** Mean and SD of the variables from 1990 to 2021.

Variable	Gender	1990	1995	2000	2005	2010	2015	2021
Alzheimer’s mortality rate per 100,000	Men	7.78 (5.11)^a^	8.61 (5.89)	9.38 (6.67)	10.40 (7.67)	11.83 (9.15)	13.68 (11.06)	15.26 (14.68)
	Women	15.12 (13.29)	16.99 (15.52)	18.66 (17.33)	20.52 (19.11)	23.06 (21.55)	26.17 (24.72)	31.56 (32.90)
	Overall	12.21 (11.43)	13.51 (13.15)	14.76 (14.74)	16.24 (16.25)	18.33 (18.33)	20.77 (20.92)	23.54 (23.82)
Percent of cardiovascular disease prevalence	Men	5.24 (2.46)	5.39 (2.51)	5.64 (2.63)	5.91 (2.78)	6.25 (2.98)	6.69 (3.21)	8.35 (3.77)
	Women	5.54 (2.92)	5.64 (2.89)	5.85 (2.96)	6.08 (3.05)	6.38 (3.17)	6.74 (3.35)	7.83 (3.51)
	Overall	5.74 (2.48)	5.94 (2.58)	6.20 (2.73)	6.51 (2.87)	6.89 (3.06)	7.35 (3.28)	8.09 (3.60)
Percent of diabetes prevalence	Men	3.13 (1.64)	3.58 (1.94)	4.21 (2.39)	4.91 (2.88)	5.68 (3.36)	6.53 (3.83)	8.11 (4.89)
	Women	3.19 (1.72)	3.62 (1.96)	4.13 (2.29)	4.80 (2.76)	5.47 (3.15)	6.24 (3.56)	7.95 (4.78)
	Overall	2.87 (1.57)	3.39 (1.93)	4.00 (2.34)	4.74 (2.82)	5.58 (3.37)	6.59 (3.97)	8.04 (4.79)
HDI	Men	62.07 (16.62)	63.77 (16.10)	65.41 (16.31)	68.28 (15.45)	70.52 (14.91)	72.74 (14.58)	73.41 (14.46)
	Women	55.87 (19.22)	58.27 (18.53)	60.14 (18.64)	63.45 (17.83)	66.31 (17.06)	69.02 (16.57)	70.05 (16.44)
	Overall	59.79 (16.38)	61.63 (16.47)	63.26 (16.86)	66.01 (16.27)	68.68 (15.58)	70.99 (15.10)	71.82 (14.97)
LEB	Men	61.83 (9.69)	62.71 (9.62)	64.16 (9.40)	65.75 (9.01)	67.50 (8.44)	68.91 (7.74)	68.54 (7.52)
	Women	67.28 (10.54)	68.06 (10.60)	69.26 (10.43)	70.82 (10.03)	72.53 (9.28)	74.03 (8.15)	73.94 (7.72)
	Overall	64.49 (10.06)	65.33 (10.04)	66.67 (9.86)	68.25 (9.46)	69.99 (8.80)	71.44 (7.90)	71.17 (7.57)
Alcohol consumption	Men	8.76 (6.86)	8.76 (6.86)	8.76 (6.86)	8.96 (6.91)	9.01 (6.68)	8.94 (6.53)	8.83 (6.39)
	Women	2.26 (1.95)	2.26 (1.95)	2.26 (1.95)	2.31 (1.97)	2.32 (1.90)	2.28 (1.83)	2.24 (1.79)
	Overall	5.42 (4.28)	5.42 (4.28)	5.42 (4.28)	5.55 (4.31)	5.58 (4.16)	5.53 (4.07)	5.46 (3.98)
Tobacco uses prevalence	Men	42.20 (15.45)	42.20 (15.45)	42.20 (15.45)	38.34 (14.71)	35.08 (14.30)	32.30 (14.14)	29.37 (13.81)
	Women	16.30 (13.15)	16.30 (13.15)	16.30 (13.15)	14.12 (11.60)	12.34 (10.48)	10.90 (9.67)	9.73 (9.26)
	Overall	29.01 (12.18)	29.01 (12.18)	29.01 (12.18)	26.01 (11.04)	23.56 (10.27)	21.54 (9.82)	19.52 (9.52)

^a^Mean (SD).

Table 2 presents the Spearman correlation coefficients among all variables for men, women, and overall data. It shows that the ADRD mortality rates exhibited strong correlations with alcohol consumption, HDI, LEB, diabetes prevalence, and particularly with cardiovascular disease prevalence. However, it's important to note that these correlations represent unadjusted relationships, which could potentially lead to misleading interpretations. For instance, the LEB might confound the relationship between ADRD mortality rates and cardiovascular prevalence, as both are significantly correlated with LEB.

**Table 2:** Spearman correlation coefficients among all variables for men, women, and the overall data.

Gender	Variables	ADRD mortality rate	Cardiovascular	Diabetes	HDI	LEB	Alcohol	Tobacco
Men	ADRD mortality rate	1						
	Cardiovascular	0.84*	1					
	Diabetes	0.63*	0.68*	1				
	HDI	0.76*	0.78*	0.78*	1			
	LEB	0.74*	0.68*	0.73*	0.92*	1		
	Alcohol	0.55*	0.52*	0.30*	0.47*	0.32*	1	
	Tobacco	0.06	0.13*	0.10*	0.04	0.03	− 0.04	1
Women	ADRD mortality rate	1						
	Cardiovascular	0.90*	1					
	Diabetes	0.63*	0.66*	1				
	HDI	0.82*	0.78*	0.75*	1			
	LEB	0.79*	0.72*	0.69*	0.93*	1		
	Alcohol	0.68*	0.62*	0.34*	0.58*	0.48*	1	
	Tobacco	0.42*	0.38*	0.27*	0.39*	0.36*	0.38*	1
Overall	ADRD mortality rate	1						
	Cardiovascular	0.90*	1					
	Diabetes	0.58*	0.61*	1				
	HDI	0.81*	0.80*	0.69*	1			
	LEB	0.78*	0.74*	0.69*	0.92*	1		
	Alcohol	0.61*	0.55*	0.24*	0.52*	0.40*	1	
	Tobacco	0.30*	0.22*	0.15*	0.23*	0.21*	0.13*	1

*Significant at 0.05 level.

In the next step, the GEE model was used to estimate the effects of time and other covariates on the mortality rate patterns of ADRD, and the results are reported in Table 3. As observed from the results of Model 1 in Table 3, the intercepts (SE) for men, women, and the overall data were 5.99 (0.29), 11.43 (0.82), and 9.61 (0.72) respectively, and the corresponding time effects (SE) were 1.25 (0.12), 2.57 (0.25), and 1.86 (0.17). These results indicate that the mean ADRD mortality rates started at 5.99, 11.43, and 9.61 individuals per 100,000 population in 1990, and increased by 1.25, 2.57, and 1.86 individuals per 100,000 every five years for men, women, and overall data, respectively. In Model 2, the results showed that the effects of cardiovascular disease (SE) were 2.31 (0.17), 5.32 (0.31), and 4.93 (0.36) for men, women, and overall data, respectively. These effects were statistically significant. This implies that for every one percent increase in cardiovascular disease prevalence, the mean ADRD mortality rates increase by 2.31, 5.32, and 4.93 persons per 100,000 population for men, women, and overall data, respectively. Although the effect of diabetes was not statistically significant for men, it had negative and significant effects for women (− 0.54) and overall data (− 0.52) in Model 2. Moreover, after adjusting for cardiovascular and diabetes covariates, the effects of time for men, women, and overall data decreased from 1.25, 2.57, and 1.86 in Model 1 to 0.17, 1.16, and 0.43 in Model 2, respectively.

**Table 3:** The effects of risk factors on ADRD mortality rates for men, women, and the overall population.

Model	Coefficients	Men	Women	Overall
1	Intercept (SE)	5.986 (0.292)*	11.429 (0.818)*	9.615 (0.717)*
	Time (SE)	1.252 (0.118)*	2.574 (0.246)*	1.859 (0.169)*
2	Intercept	− 4.334 (0.844)*	− 13.668 (1.620)*	− 14.997 (1.780)*
	Time	0.171 (0.078)*	1.160 (0.233)*	0.433 (0.129)*
	Cardiovascular	2.311 (0.170)*	5.322 (0.308)*	4.933 (0.364)*
	Diabetes	0.057 (0.105)	− 0.543 (0.217)*	− 0.520 (0.183)*
3	Intercept	− 15.766 (2.503)*	− 22.039 (5.548)*	− 23.800 (4.109)*
	Time	0.101 (0.095)	0.994 (0.312)*	0.283 (0.174)
	Cardiovascular	2.024 (0.205)*	4.741 (0.451)*	4.995 (0.526)*
	Diabetes	0.259 (0.157)	− 0.261 (0.354)	− 0.320 (0.259)
	HDI	− 0.060 (0.057)	0.231 (0.120)	− 0.034 (0.121)
	LEB	0.250 (0.075)*	− 0.053 (0.137)	0.145 (0.124)
4	Intercept	− 11.764 (2.475)*	− 25.526 (5.826)*	− 18.563 (4.162)*
	Time	− 0.176 (0.101)	1.240 (0.325)*	− 0.229 (0.212)
	Cardiovascular	2.222 (0.240)*	4.846 (0.629)*	5.405 (0.636)*
	Diabetes	0.323 (0.167)	− 0.103 (0.385)	− 0.045 (0.313)
	HDI	− 0.084 (0.058)	0.172 (0.119)	− 0.057 (0.133)
	LEB	0.273 (0.077)*	0.010 (0.142)	0.181 (0.133)
	Alcohol	− 0.057 (0.062)	− 0.245 (0.569)	− 0.625 (0.190)*
	Tobacco	− 0.103 (0.024)*	0.084 (0.082)	− 0.164 (0.068)
5	Intercept	− 39.490 (7.746)*	− 6.835 (8.092)	− 22.639 (7.531)*
	Time	− 0.087 (0.111)	0.956 (0.317)*	− 0.271 (0.198)
	Cardiovascular	1.845 (0.249)*	3.945 (0.732)*	4.700 (0.701)*
	Diabetes	0.261 (0.158)	0.207 (0.589)	0.030 (0.311)
	HDI	− 0.087 (0.064)	− 0.046 (0.106)	0.096 (0.111)
	LEB	0.731 (0.135)*	− 0.004 (0.139)	0.137 (0.127)
	Alcohol	− 0.593 (0.289)*	− 9.916 (2.583)*	− 2.316 (0.773)*
	Alcohol * HDI	0.009 (0.004)*	0.154 (0.036)*	0.029 (0.012)*
	Tobacco	0.922 (0.157)*	0.134 (0.163)	0.392 (0.125)*
	Tobacco * HDI	− 0.016 (0.003)*	− 0.002 (0.003)	− 0.009 (0.002)*

*Significant at 0.05 level.

In Model 3, we included the effects of HDI and LEB, and the results are also reported in Table 3. We found that these effects were not significant for women and overall data. However, for men, the effect of LEB was significant and equal to 0.25. In Model 4, we included the effects of alcohol and tobacco use. The effect of alcohol was − 0.625 for the overall data, and the effect of tobacco was − 0.103 for men. Both of these effects were negative and statistically significant at the 0.05 level. These results indicate that for every one-liter increase in pure alcohol consumption among the overall population and a one percent increase in the prevalence of tobacco use among men, the mean ADRD death rates decreased by 0.625 and 0.103 individuals per 100,000 population, respectively.

In Model 5, we examined the interaction effects of alcohol with HDI. For men, women, and overall data, the interaction effects were 0.01, 0.15, and 0.03, respectively. Additionally, the main effects of alcohol were − 0.59, − 9.92, and − 2.32 for men, women, and overall data, respectively. All of these effects were statistically significant. These findings indicate that while the main effect of alcohol consumption on the ADRD mortality rates was negative, it significantly depended on the HDI index. Specifically, the interaction effect suggests that an increase in alcohol consumption in countries with high HDI could potentially have a lower effect on decreasing ADRD mortality rates. On the other hand, for men and the overall data, we observed interaction coefficients of − 0.02 and − 0.01 between tobacco and HDI, while the main effects of tobacco were 0.92 and 0.39, respectively. All of these effects were statistically significant at the 0.05 level. These findings suggest that while an increase in tobacco use can lead to an increase in ADRD mortality rate, this effect depends on the HDI index. Specifically, increasing the HDI can mitigate the effect of tobacco on the ADRD death rate.

**Figure 1:**
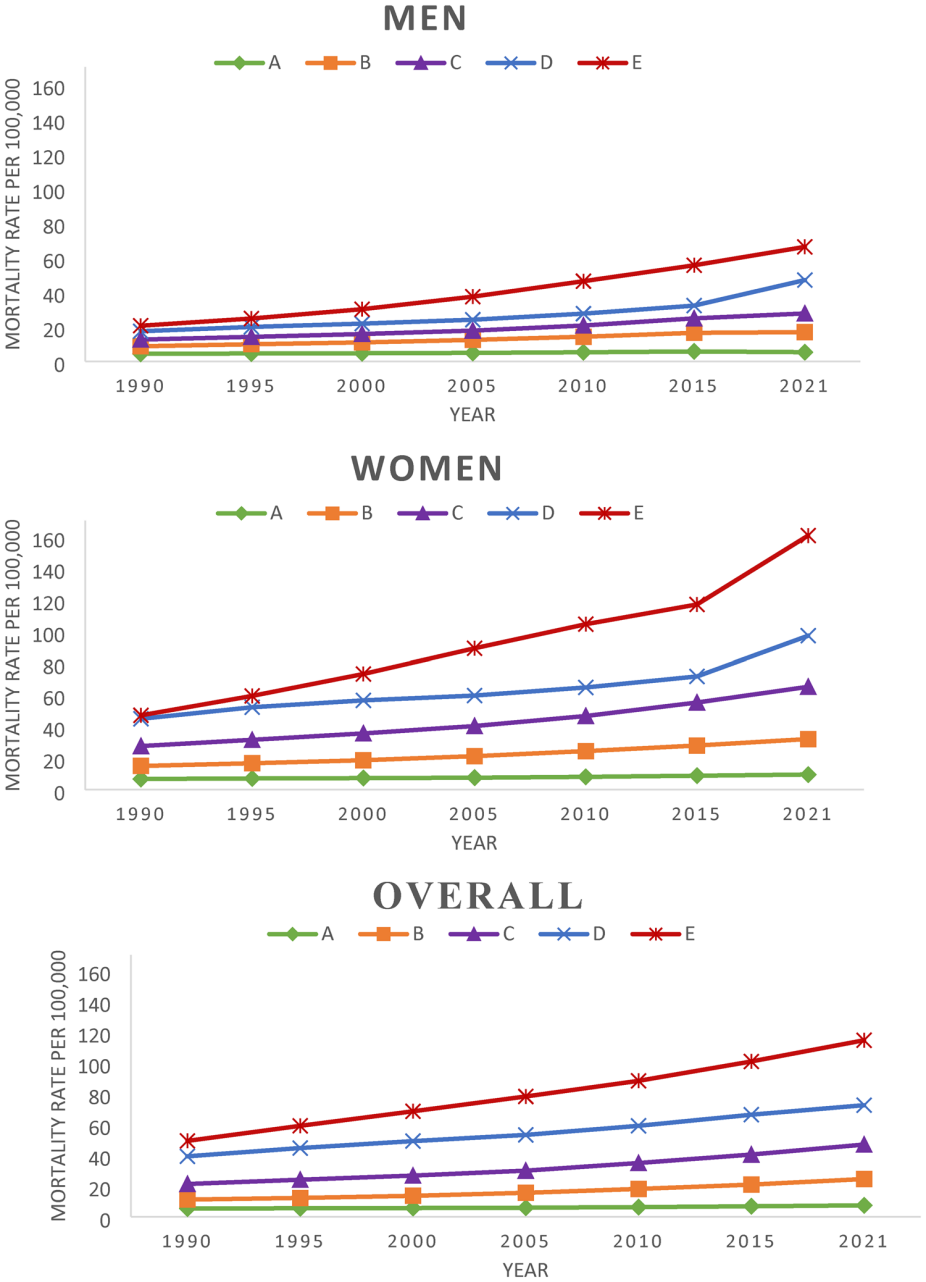
The average ADRD mortality rates for each cluster among men, women, and the overall population.

In the next step, we employed a longitudinal k-means clustering approach on the overall data to categorize countries based on their patterns of ADRD mortality rates. We applied the same clusters, which were derived from the analysis of the overall data, to both men and women. This allowed us to compare the effects of risk factors between the two genders. The analysis revealed that the model with five clusters had the highest Calinski–Harabasz index value, enabling us to categorize our sample into five distinct groups. The cluster patterns for men, women, and the overall data are presented in Fig. 1. Among all the clusters, cluster A exhibited the lowest mortality rate with a slower-increasing pattern. The mean initial values and slopes of the ADRD mortality rates increased for clusters B, C, D, and E, respectively. It is worth noting that cluster E, which consisted of only three countries (Japan, Italy, and Monaco), had the highest mortality rate at the beginning and demonstrated the steepest increasing pattern.

**Figure 2:**
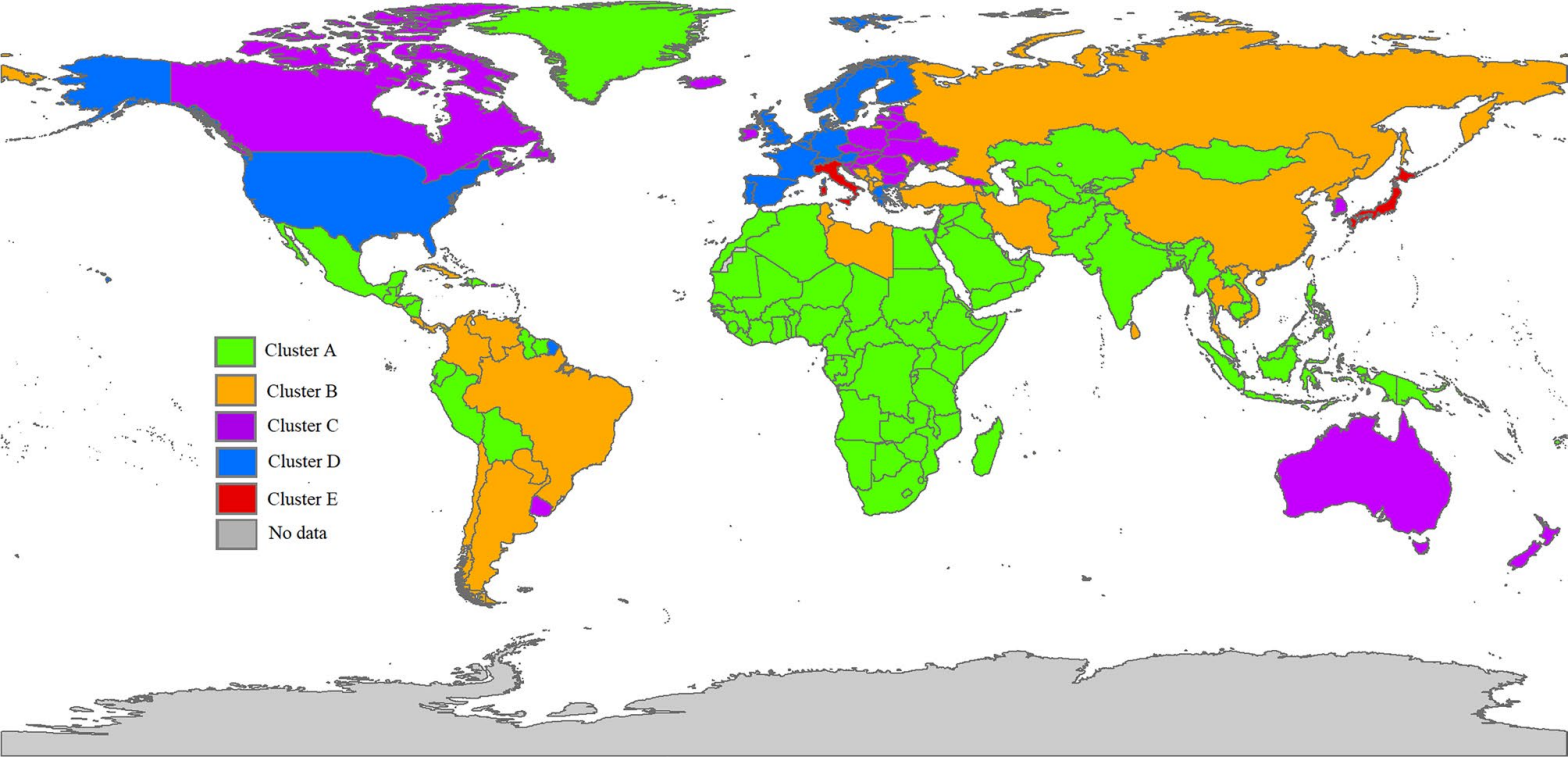
The geographic distribution of all 204 countries based on their membership in different clusters.

In the next step, the GEE model was used to estimate the effects of risk factors for each class, with class E being excluded due to its limited sample size. The results of the GEE model for the four clusters for men, women, and overall data are presented in Table 4. In Model 1, it was observed that the time effect was positive and significant for all four clusters in men, women, and overall data. Among the clusters, cluster A exhibited the lowest time effect, while cluster D demonstrated the highest time effect. In Model 2, the effect of cardiovascular disease prevalence was found to be significant and positive for all clusters in men, women, and overall data. However, the effect of diabetes in cluster B was significantly negative for the overall data. Moving on to Model 3, we observed that the effect of HDI was positive for clusters B and D in the overall data. However, in cluster C, it had a negative effect for men, while in cluster B, it had a positive effect for women. These effects were statistically significant at the 0.05 level. Additionally, in Model 3, we found that LEB had a significant positive effect in cluster C for the overall data. It also had a positive effect in cluster B and C for men, and a positive effect in cluster D for women. In Model 4, we observed that the effect of alcohol consumption was significantly negative in cluster A of the overall data and cluster D of the men's data. However, it was non-significant for women. In addition, in Model 4, we found that the effect of tobacco use was statistically significant in cluster A of the overall data. Furthermore, its effect was significantly negative in clusters B and C for men. Figure 2 and Table 5 present the geographic distribution of all 204 countries and territories categorized into different clusters.

**Table 4:** The results of the GEE model for each cluster for men, women, and the overall population.

Gender	Model	Coefficient	Cluster A	Cluster B	Cluster C	Cluster D
Men	1	Intercept (SE)	4.137 (0.204)*	6.935 (0.632)*	8.831 (0.655)*	10.104 (1.367)*
		Time (SE)	0.206 (0.041)*	1.462 (0.139)*	2.558 (0.181)*	4.255 (0.361)*
	2	Intercept (SE)	1.947 (0.488)*	3.675 (1.142)*	2.122 (1.758)	− 12.341 (7.530)
		Time (SE)	− 0.026 (0.039)	0.920 (0.172)*	1.714 (0.280)*	2.518 (0.560)*
		Cardiovascular (SE)	0.649 (0.143)*	0.828 (0.232)*	0.834 (0.266)*	2.440 (0.745)*
		Diabetes (SE)	0.102 (0.055)	− 0.020 (0.118)	0.291 (0.316)	0.363 (0.407)
	3	Intercept (SE)	0.476 (0.834)	− 23.826 (6.617)*	− 26.913 (5.098)*	59.894 85.673
		Time (SE)	− 0.089 (0.048)	0.727 (0.208)*	1.498 (0.242)*	4.099 (1.902)*
		Cardiovascular (SE)	0.517 (0.177)*	0.642 (0.210)*	1.081 (0.177)*	1.807 (0.730)*
		Diabetes (SE)	0.114 (0.084)	− 0.081 (0.139)	0.294 (0.163)	0.237 (0.450)
		HDI (SE)	− 0.001 (0.025)	0.109 (0.077)	− 0.370 (0.102)*	− 0.338 (0.222)
		LEB (SE)	0.041 (0.023)	0.315 (0.123)*	0.797 (0.137)*	− 0.536 (1.116)
	4	Intercept (SE)	− 0.538 (0.916)	− 20.122 (7.462)*	− 5.223 (9.587)	154.006 (101.939)
		Time (SE)	− 0.042 (0.057)	0.383 (0.290)	1.499 (0.225)*	4.424 (1.860)*
		Cardiovascular (SE)	0.475 (0.213)*	0.752 (0.206)*	1.022 (0.144)*	1.648 (0.777)*
		Diabetes (SE)	0.068 (0.088)	− 0.035 (0.158)	0.257 (0.167)	0.131 (0.528)
		HDI (SE)	0.010 (0.025)	0.117 (0.103)	− 0.353 (0.097)*	− 0.160 (0.200)
		LEB (SE)	0.037 (0.023)	0.316 (0.148)*	0.560 (0.169)*	− 1.659 (1.295)
		Alcohol (SE)	0.035 (0.027)	− 0.034 (0.082)	− 0.050 (0.071)	− 1.103 (0.454)*
		Tobacco (SE)	0.013 (0.010)	− 0.087 (0.037)*	− 0.109 (0.033)*	− 0.116 (0.110)
Women	1	Intercept (SE)	6.067 (0.369)*	10.902 (1.186)*	18.696 (1.846)*	34.585 (3.418)*
		Time (SE)	0.449 (0.062)*	2.836 (0.241)*	6.104 (0.437)*	7.304 (0.672)*
	2	Intercept (SE)	− 0.680 (0.752)	1.586 (1.736)	4.253 (4.580)	− 13.471 (14.877)
		Time (SE)	0.053 (0.061)	1.925 (0.290)*	5.300 (0.505)*	6.588 (0.616)*
		Cardiovascular (SE)	2.100 (0.236)*	2.351 (0.383)*	1.800 (0.453)*	4.715 (1.314)*
		Diabetes (SE)	− 0.103 (0.080)	− 0.446 (0.228)	− 0.138 (0.739)	− 0.184 (0.944)
	3	Intercept (SE)	− 3.248 (1.336)*	− 1.506 (15.214)	− 80.081 (30.989)*	− 258.914 (120.852)*
		Time (SE)	− 0.111 (0.072)	1.900 (0.390)*	3.326 (0.655)*	4.123 (1.596)*
		Cardiovascular (SE)	1.894 (0.261)*	1.511 (0.266)*	2.300 (0.525)*	5.260 (1.474)*
		Diabetes (SE)	− 0.123 (0.100)	− 0.514 (0.208)*	1.277 (0.578)*	0.049 (0.650)
		HDI (SE)	0.046 (0.026)	0.367 (0.136)*	0.049 (0.230)	− 0.145 (0.311)
		LEB (SE)	0.028 (0.026)	− 0.220 (0.280)	0.943 (0.485)	3.179 (1.579)*
	4	Intercept (SE)	− 3.833 (1.497)*	17.721 (19.576)	− 74.978 (32.041)*	− 223.986 (117.016)
		Time (SE)	− 0.103 (0.081)	1.868 (0.562)*	3.032 (0.671)*	4.999 (1.257)*
		Cardiovascular (SE)	2.035 (0.305)*	1.363 (0.362)*	2.470 (0.591)*	5.150 (1.621)*
		Diabetes (SE)	− 0.133 (0.102)	− 0.347 (0.208)	1.271 (0.575)*	0.041 (0.735)
		HDI (SE)	0.020 (0.027)	0.478 (0.197)*	0.110 (0.221)	− 0.206 (0.313)
		LEB (SE)	0.047 (0.027)	− 0.550 (0.365)	0.859 (0.480)	2.725 (1.529)
		Alcohol (SE)	0.206 (0.209)	− 0.516 (0.630)	− 0.413 (0.819)	0.261 (1.471)
		Tobacco (SE)	− 0.030 (0.017)	0.036 (0.066)	− 0.096 (0.130)	0.156 (0.262)
Overall	1	Intercept (SE)	4.810 (0.226)*	7.737 (0.675)*	15.220 (1.17)*	33.027 (2.455)*
		Time (SE)	0.330 (0.048)*	2.210 (0.182)*	4.206 (0.286)*	5.461 (0.545)*
	2	Intercept (SE)	− 2.473 (0.669)*	− 5.514 (1.937)*	− 1.777 (3.724)	1.408 (10.215)
		Time (SE)	0.030 (0.034)	0.859 (0.258)*	2.742 (0.475)*	3.164 (0.892)*
		Cardiovascular (SE)	1.916 (0.177)*	2.991 (0.423)*	2.248 (0.641)*	2.737 (0.922)*
		Diabetes (SE)	− 0.108 (0.066)	− 0.451 (0.223)*	0.060 (0.770)	1.336 (1.131)
	3	Intercept (SE)	− 3.349 (1.007)*	− 7.347 (7.708)	− 39.068 (13.078)*	− 37.305 (142.270)
		Time (SE)	− 0.030 (0.046)	1.142 (0.270)*	1.308 (0.473)*	1.589 (2.039)
		Cardiovascular (SE)	1.807 (0.185)*	2.216 (0.268)*	2.426 (0.468)*	2.699 (0.804)*
		Diabetes (SE)	− 0.123 (0.069)	− 0.643 (0.200)*	1.342 (0.324)*	1.711 (0.987)
		HDI (SE)	0.025 (0.022)	0.229 (0.102)*	− 0.045 (0.154)	0.789 (0.195)*
		LEB (SE)	0.005 (0.019)	− 0.130 (0.154)	0.511 (0.189)*	− 0.330 (1.993)
	4	Intercept (SE)	− 2.139 (1.286)	− 0.156 (8.922)	− 25.963 (17.729)	11.201 (144.466)
		Time (SE)	− 0.023 (0.057)	0.973 (0.360)*	0.950 (0.512)	1.837 (1.574)
		Cardiovascular (SE)	1.876 (0.217)*	2.345 (0.317)*	2.486 (0.511)*	2.639 (0.822)*
		Diabetes (SE)	− 0.126 (0.086)	− 0.398 (0.227)	1.244 (0.372)*	1.480 (0.935)
		HDI (SE)	− 0.010 (0.024)	0.264 (0.101)*	0.038 (0.144)	0.843 (0.239)*
		LEB (SE)	0.023 (0.020)	− 0.247 (0.171)	0.368 (0.213)	− 0.784 (2.018)
		Alcohol (SE)	− 0.005 (0.055)	− 0.472 (0.139)*	− 0.249 (0.242)	− 1.619 (0.979)
		Tobacco (SE)	− 0.035 (0.014)*	0.027 (0.058)	− 0.168 (0.123)	0.033 (0.179)

*Significant at 0.05 level.

**Table 5:** Cluster membership of each country.

Cluster	Countries
A	Afghanistan, Algeria, American Samoa, Angola, Azerbaijan, Bahamas, Bahrain, Bangladesh, Bhutan, Bolivia, Botswana, Belize, Solomon Islands, Brunei Darussalam, Myanmar, Burundi, Cambodia, Cameroon, Central African Republic, Chad, Comoros, Congo, Democratic Republic of the Congo, Benin, Dominican Republic, Ecuador, Equatorial Guinea, Ethiopia, Eritrea, Fiji, Djibouti, Gabon, Gambia, Palestine, Ghana, Kiribati, Greenland, Guatemala, Guinea, Guyana, Haiti, Honduras, India, Indonesia, Iraq, Coote d'Ivoire, Kazakhstan, Jordan, Kenya, Kuwait, Kyrgyzstan, Lao People's Democratic Republic, Lesotho, Liberia, Madagascar, Malawi, Malaysia, Maldives, Mali, Mauritania, Mexico, Mongolia, Mozambique, Oman, Namibia, Nauru, Nepal, Vanuatu, Nicaragua, Niger, Nigeria, Northern Mariana Islands, Micronesia (Federated States of), Marshall Islands, Palau, Pakistan, Papua New Guinea, Peru, Philippines, Guinea-Bissau, Timor-Leste, Qatar, Rwanda, Saint Kitts and Nevis, Sao Tome and Principe, Saudi Arabia, Senegal, Sierra Leone, Somalia, South Africa, Zimbabwe, South Sudan, Sudan, Suriname, Eswatini, Syrian Arab Republic, Tajikistan, Togo, United Arab Emirates, Turkmenistan, Tuvalu, Uganda, Egypt, United Republic of Tanzania, Burkina Faso, Uzbekistan, Samoa, Yemen, Zambia
B	Albania, Antigua and Barbuda, Argentina, Armenia, Barbados, Bosnia and Herzegovina, Brazil, Cabo Verde, Sri Lanka, Chile, China, Taiwan (Province of China), Colombia, Cook Islands, Costa Rica, Cuba, Cyprus, Dominica, El Salvador, Grenada, Guam, Iran (Islamic Republic of), Jamaica, Democratic People's Republic of Korea, Lebanon, Libya, Mauritius, Republic of Moldova, Montenegro, Morocco, Panama, Paraguay, Russian Federation, Saint Lucia, Saint Vincent and the Grenadines, Serbia, Seychelles, Singapore, Viet Nam, Thailand, Tokelau, Tonga, Trinidad and Tobago, Tunisia, Turkey, North Macedonia, United States Virgin Islands, Venezuela
C	Andorra, Australia, Bermuda, Bulgaria, Belarus, Canada, Croatia, Czechia, Estonia, Georgia, Hungary, Iceland, Ireland, Israel, Republic of Korea, Latvia, Lithuania, Luxembourg, Malta, New Zealand, Niue, Poland, Puerto Rico, Romania, Slovakia, Slovenia, Ukraine, Uruguay
D	Austria, Belgium, Denmark, Finland, France, Germany, Greece, Netherlands, Norway, Portugal, San Marino, Spain, Sweden, Switzerland, United Kingdom, United States of America
E	Italy, Japan, Monaco

## Discussion

In this study, the GEE model was utilized to estimate the effects of risk factors on the mortality rates of Alzheimer's Disease and Related Dementias (ADRD) worldwide from 1990 to 2021, specifically focusing on men, women, and the overall population. Subsequently, the data were clustered into five categories using a longitudinal k-means clustering approach based on the overall ADRD mortality rate. The GEE model was then applied to estimate the effects of risk factors on the mortality rate patterns within each cluster. The average mortality rates of ADRD almost doubled over the studied period, with rates increasing from 7.78, 15.12, and 12.21 per 100,000 in 1990 to 15.26, 31.56, and 23.54 per 100,000 in 2021 for men, women, and overall data, respectively. According to Lie et al., the number of global deaths attributed to ADRD increased from 0.56 million in 1990 to 1.62 million in 2019, nearly tripling over a span of 30 years^4^. Furthermore, women had a nearly two times higher rates compared to men^4^. In another study, it was found that the number of deaths due to ADRD increased by almost 2.5 times between 1990 and 2016^25^. This worldwide increase in mortality could be attributed to the increase in lifespan and population growth. Additionally, the higher mortality rates among women may be influenced by their longer life expectancy compared to men.

The effects of cardiovascular disease prevalence on the death rates of ADRD were statistically significant for men, women, and the overall population. However, women demonstrated a higher effect of cardiovascular disease prevalence on the ADRD mortality rates compared to men. According to Tini et al., the association between gender differences and cardiovascular risk factors for Alzheimer's Disease (AD) suggests that while conditions such as hypertension, high cholesterol, and diabetes are linked to an increased risk of developing both cardiovascular disease and AD in both women and men, women with these risk factors appear to face a higher risk of developing AD compared to men^26^. Some studies have investigated the relationship between ADRD and cardiovascular disease, revealing that cardiovascular risk factors are closely associated with the development of ADRD^26,27^. Clinical studies have shown that cardiovascular disease and dementia share similar genetic and biochemical profiles as well as common triggers^26^. The main benefit of this association is that it shows a potential opportunity to prevent dementia by effectively managing and treating cardiovascular disease and its risk factors^28^. This involves implementing both drug-based treatments and lifestyle adjustments that aim to enhance cardiovascular health. Moreover, in Model 5 as presented in Table 3, the increasing prevalence of diabetes was found to be associated with a rise in ADRD mortality rates. However, this association was not statistically significant. A quantitative meta-analysis of 19 studies showed that individuals with diabetes had a higher risk of dementia compared to healthy controls^29^.

Based on the results from Table 3 in Model 5, the effect of tobacco was positive, while its interaction with HDI was negative. These findings suggest that while an increase in tobacco use can lead to an increase in ADRD mortality rates, this effect depends on the HDI index. More precisely, elevating the HDI can alleviate the impact of tobacco on the mortality rates associated with ADRD. A meta-analysis of 37 studies revealed that individuals who currently smoke consistently exhibited higher risks of all-cause dementia, Alzheimer’s disease, and vascular dementia when compared to those who never smoked^30^. However, this study did not find elevated risks of dementia among former smokers^30^. It is important to note that the effect of tobacco on ADRD mortality rates was stronger and statistically significant for men compared to women. This difference may be attributed to the fact that men tend to engage in excessive smoking more than women.

The effect of alcohol was found to be negative, and its interaction with HDI was positive for men, women, and overall data. This suggests that an increase in alcohol consumption can potentially reduce ADRD mortality rates. However, higher HDI levels could reduce the protective effect of alcohol consumption. Our analysis showed that countries with higher HDI tend to have higher levels of alcohol consumption (Spearman correlation coefficient: men, r = 0.47, p < 0.05; women, r = 0.58, p < 0.05; overall, r = 0.52, p < 0.05). This suggests that countries with high HDI should consider reducing their alcohol consumption to decrease their ADRD mortality rates. Notably, the effect of alcohol consumption was found to be more protective for women compared to men. This difference may be attributed to the fact that women generally have lower levels of alcohol consumption than men. In other words, excessive alcohol drinking could potentially reduce the protective effect of alcohol on ADRD mortality rates. Many studies have commonly suggested that light-to-moderate alcohol intake might have a protective effect against dementia, while excessive drinking may instead increase the risk^31–33^.

The longitudinal k-means clustering method classified countries into five distinct groups based on their overall growth trajectories. The geographic distribution map of all 204 countries and territories is shown in Fig. 2 and Table 5. Cluster A consisted mostly of African and Middle Eastern countries, as well as some developing nations in Eastern Asia and Latin America. In Cluster A, according to the GEE results, cardiovascular disease was significantly associated with increased mortality rates of ADRD, while tobacco exhibited a protective effect. It is important to note that the impact of cardiovascular disease on the mortality rates of ADRD remained statistically significant across all clusters, even after adjusting for other risk factors such as HDI, LEB, alcohol consumption, tobacco use, and diabetes in Model 4, as presented in Table 4. As mentioned earlier, previous studies indicated that cardiovascular risk factors are closely associated with the development of dementia^26,27^. A survey conducted in Latin America, China, and India revealed that in these countries, there is a higher likelihood of dementia among individuals with a history of tobacco smoking, whereas no significant association was found with the use of smokeless tobacco^34^. It is worth mentioning that the effect of tobacco was found to be protective in Cluster A for overall data and in Clusters B and C for men. This finding is inconsistent with other studies that have either shown a positive association or no significant relationship between smoking and the onset of any ADRD^34–36^. The observed protective effect of tobacco may be attributed to a potential confounding effect of life expectancy. Countries with high tobacco use tend to have lower life expectancy, which may lead to lower ADRD death rates^37^.

Cluster B comprised countries such as Russia, Libya, Iran, Tunisia, Turkey, Lebanon, Albania, Bosnia and Herzegovina, Cyprus, Serbia, most countries in Latin America, and some Eastern Asian countries including China, North Korea, Vietnam, Singapore, Taiwan, and Thailand. Alcohol consumption had a protective effect in all clusters, particularly in cluster B, which was statistically significant. Some studies have indicated that moderate alcohol consumption might be linked to a decrease in the mortality rate related to ADRD^31–33^. In cluster B, an association was observed between HDI and increasing ADRD mortality rates, which may be attributed to the higher life expectancy in these countries. This association suggests that countries with higher HDI, which typically have higher life expectancy, also tend to have higher ADRD death rates.

Cluster C consisted of countries such as Canada, the Republic of Korea, Australia, New Zealand, Puerto Rico, Uruguay, Israel, Ireland, and some Central European countries. These countries exhibited higher mortality rates with a greater rate of increase compared to countries in clusters A and B. In cluster C, an increase in diabetes was found to be associated with a rise in the ADRD mortality rates. This finding aligns with previous research studies that have shown a significant association between diabetes and dementia^38,39^. It was concluded that better diabetes treatment has the potential to decrease the risk of ADRD in the coming decades^40^.

Cluster D, which included European countries and the United States of America, had higher initial death rates and a higher rate of increase compared to clusters A, B, and C. In cluster D, it was observed that both the prevalence of cardiovascular disease and HDI were associated with higher mortality rates of ADRD. Some studies have examined the relationship between ADRD and cardiovascular disease, indicating a close association between cardiovascular risk factors and the development of ADRD^26,27^. Additionally, HDI is associated with life expectancy, and as a result, higher life expectancy could increase the ADRD mortality rate.

Cluster E, comprising only Japan, Italy, and Monaco, had the highest ADRD mortality rates. Based on our results, the burden of ADRD in these countries is substantial and continues to grow. According to the 2020 data report published by the WHO, dementia deaths in Italy ranked as the fifth leading cause^41^. Italy, known for having one of the oldest populations in the world, and Japan and Monaco, known for having the highest life expectancy in the world, are witnessing an increasing number of older individuals in their populations who are at risk of developing dementia^41–43^.

## Strengths and limitations

The main strength of this study was its utilization of the longitudinal k-means clustering method to categorize countries into homogeneous subgroups, followed by the use of the GEE model to estimate the effects of risk factors on ADRD mortality rates within each subgroup. Furthermore, the inclusion of interaction terms enhanced the explanation of risk factor effects over time. However, it is important to acknowledge several limitations of the study. First, the quality and accuracy of the data obtained from the GBD platform varied across countries. The GBD utilizes diverse sources, including censuses, household surveys, civil registration and vital statistics, disease registries, health service use, air pollution monitors, satellite imaging, disease notifications, and other relevant data^44^. Through the application of modeling techniques, the GBD estimates mortality rates^44^. The variability in the multiple sources used for data collection and estimation could potentially introduce biases and impact the generalizability of the findings. Second, there were missing data in covariates such as smoking, alcohol, HDI, and LEB, which were obtained from the WHO and UNDP platforms. As a result, samples with missing data had to be excluded from the GEE model analysis, leading to a reduction in the overall sample size. This reduction in sample size could potentially impact the statistical power of the analysis. Finally, there were significant correlations among some of the covariates, which could result in multicollinearity and affect the estimation of parameters. Furthermore, caution should be exercised when interpreting the covariates, as there may be additional confounding factors that exist and can impact the results. It is important to emphasize that the presence of an association does not necessarily indicate a causal relationship.

## Conclusion

In general, both cardiovascular disease and tobacco use have been associated with an increase in total mortality rates related to ADRD, while alcohol consumption has had a protective effect on the pattern of ADRD mortality rates. It is important to note that the effects of tobacco use and alcohol consumption on ADRD mortality rates were significantly dependent on the HDI. In addition, the impact of tobacco use was stronger among men compared to women, while the protective effect of alcohol consumption was more prominent among women compared to men. However, there was heterogeneity in the pattern of ADRD mortality rates across the world, and the 204 countries and territories were classified into five clusters with different growth trajectories. Underdeveloped and developing countries with low death rates and low increasing patterns of mortality rates were classified into clusters A and B. Developed countries, which had higher mortality rates with higher increasing patterns, were categorized into clusters C, D, and E. The effect of tobacco in cluster A was found to be protective, and similarly, the effect of alcohol in cluster B also showed a protective effect. These findings indicate the need for further investigation to identify the underlying causal factors and possible confounding factors in these clusters. In European countries within cluster D, the increasing prevalence of diabetes was associated with a rise in ADRD mortality rates. Diabetes can be effectively managed or delayed through interventions such as adopting a healthy diet, engaging in regular physical activity, and using appropriate medications. Furthermore, it was observed that the rising prevalence of cardiovascular disease in all clusters was associated with increasing mortality rates of ADRD. The significant benefit of this association is that it highlights a potential opportunity to prevent ADRD by effectively managing and treating cardiovascular disease and its risk factors.

## Data Availability

All of the data in this study was obtained from freely accessible online databases, including the Global Burden of Disease (GBD), the World Health Organization (WHO), and the United Nations Development Programme (UNDP)^17,18,21,22^.

## Abbreviations

ADRD: Alzheimer’s disease and related dementias
GBD: Global Burden of Disease
WHO: World Health Organization
UNDP: United Nations Development Programme
HDI: Human development index
LEB: Life expectancy at birth
GEE: Generalized estimating equations
GNI: Gross national income
SD: Standard deviation
SE: Standard error
